# Genetically proxied antidiabetic drugs targets and stroke risk

**DOI:** 10.1186/s12967-023-04565-x

**Published:** 2023-09-30

**Authors:** Yahui Zhu, Mao Li, Hongfen Wang, Fei Yang, Xinyuan Pang, Rongrong Du, Jinghong Zhang, Xusheng Huang

**Affiliations:** 1 Medical School of Chinese PLA, Beijing, China; 2 Department of Neurology, The First Medical Center, Chinese PLA General Hospital, No. 28 Fuxing Road, Haidian District, Beijing, 100853 China; 3 College of Medicine, Nankai University, Tianjin, China

**Keywords:** Antidiabetic drugs, Stroke, Mendelian randomization, Sulfonylurea

## Abstract

**Background:**

Previous studies have assessed the association between antidiabetic drugs and stroke risk, but the results are inconsistent. Mendelian randomization (MR) was used to assess effects of antidiabetic drugs on stroke risk.

**Methods:**

We selected blood glucose-lowering variants in genes encoding antidiabetic drugs targets from genome-wide association studies (GWAS). A two-sample MR and Colocalization analyses were applied to examine associations between antidiabetic drugs and the risk of stroke. For antidiabetic agents that had effect on stroke risk, an independent blood glucose GWAS summary data was used for further verification.

**Results:**

Genetic proxies for sulfonylureas targets were associated with reduced risk of any stroke (OR=0.062, 95% CI 0.013-0.295, P=4.65×10^－4^) and any ischemic stroke (OR=0.055, 95% CI 0.010-0.289, P=6.25×10^－4^), but not with intracranial hemorrhage. Colocalization supported shared casual variants for blood glucose with any stroke and any ischemic stroke within the encoding genes for sulfonylureas targets (KCNJ11 and ABCC8) (posterior probability>0.7). Furthermore, genetic variants in the targets of insulin/insulin analogues, glucagon-like peptide-1 analogues, thiazolidinediones, and metformin were not associated with the risk of any stroke, any ischemic stroke and intracranial hemorrhage. The association was consistent in the analysis of sulfonylureas with stroke risk using an independent blood glucose GWAS summary data.

**Conclusions:**

Our findings showed that genetic proxies for sulfonylureas targets by lowering blood glucose were associated with a lower risk of any stroke and any ischemic stroke. The study might be of great significance to guide the selection of glucose-lowering drugs in individuals at high risk of stroke.

**Supplementary Information:**

The online version contains supplementary material available at 10.1186/s12967-023-04565-x.

## Background

Stroke is one of the leading causes of death and disability worldwide [1, 2]. Diabetes is associated with a substantially increased risk of stroke. A meta-analysis of the prospective studies showed that the hazard ratio of ischemic stroke and haemorrhagic stroke in patients with diabetes was 2.27 and 1.56 compared with those without diabetes [3], and at the population level, diabetes may lead to > 8% of the first ischemic stroke [4]. In addition, stroke outcomes in diabetic patients are worse, leading to increased mortality and disability [4]. Therefore, the way to prevent stroke in these patients has become an important issue. Most diabetic patients control blood glucose by taking antidiabetic drugs. Although most major trials of antidiabetic drugs have focused on glucose-lowering properties, it is crucial to study whether an anti-diabetic drug has a protective effect against stroke, and whether one drug is better than another.

Previous studies have evaluated the effect of antidiabetic drugs on stroke risk. The accumulated evidence suggested that metformin, pioglitazone and semaglutide reduce stroke risk [5]. A meta-analysis using data from randomized clinical trials of antidiabetic drugs was conducted to study the association between glucose-lowering treatment and the risk of stroke [6]. The results showed that glucagon-like peptide-1 receptor (GLP-1) agonists and thiazolidinediones (TZD) reduced the risk of stroke, while sulfonylureas (SU), dipeptidyl peptidase-4 (DPP-4) inhibitors, sodium-glucose cotransporter 2 (SGLT2) inhibitors, alpha-glucosidase inhibitors, meglitinides, and metformin had no effect on stroke risk. Although these trials assessed the effect of antidiabetic drugs on stroke risk, the results of these trials must be carefully compared and contrasted given the differences in the enrolled populations and trial design.

Mendelian randomization (MR), which uses genetic variants as proxies for traits of interest, is used to assess causal association. MR is less susceptible to confounders and can avoid reverse causality compared with observational studies [7, 8]. Here, we used genetic data on blood glucose and stroke to examine the effects of genetic proxies for antidiabetic drugs targets on stroke risk using MR.

## Methods

**Fig. 1:**
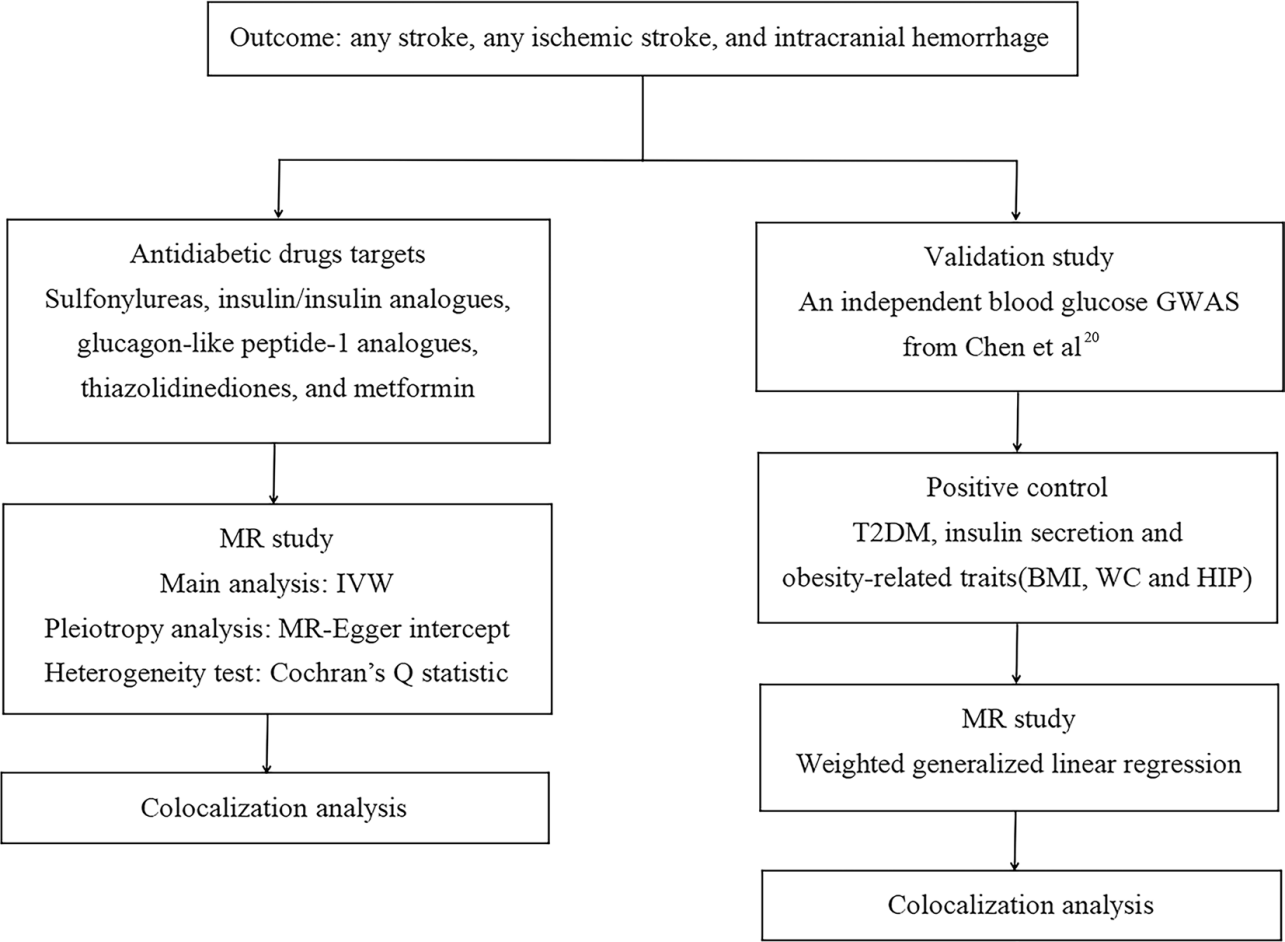
A flow diagram summarizing the study. MR, Mendelian randomization; IVW, inverse variance weighted; GWAS, genome-wide association study; T2DM, type 2 diabetes mellitus; BMI, body mass index; WC, waist circumference; HIP, hip circumference

This study followed the Strengthening the Reporting of Observational Studies in Epidemiology Using Mendelian Randomization (STROBE-MR) guide [9]. A flow diagram summarizing the study was shown in Fig. 1.

### Genetic instrument selection

Five major classes of antidiabetic drugs were initially identified, including sulfonylureas, insulin/insulin analogues, GLP-1 analogues, thiazolidinediones and metformin [10]. Genetic variants for drug targets of sulfonylureas, insulin/insulin analogues, GLP-1 analogues, and thiazolidinediones were selected according to the method reported by Tang et al. using blood glucose GWAS summary data in 326,885 participants of European ancestry from UK Biobank [11]. In the study of Tang et al., rs757110, which has been validated as a strong proxy of sulfonylureas in previous studies, was chosen as an additional variant of sulfonylureas. In short, the cis-variants within each encoding gene of drug target (± 2500 base pairs of the gene location) were identified and the variants associated with blood glucose at a false discovery rate of < 0.05 were retained. Palindromic single-nucleotide variations were excluded to avoid ambiguity in the identification of effect alleles. The variants were then clumped with a R^2^ of 0.01 and a window size of 500 kB.

In addition, genetic variants for drug targets of metformin were selected according to the method reported by Zheng et al. using glycated hemoglobin (HbA1c) GWAS summary data in 415,576 individuals of European ancestry from UK Biobank [12]. In short, drug targets and encoding genes of metformin were identified. Genetic variants near each of the metformin genes that associated with both the glycaemic biomarker HbA1c and the expression level of the corresponding gene were selected. After validation, the genetic predictors for each target were generated, with effects quantified as the HbA1c-lowering effect of the target.

Information for the GWAS datasets used in our study was summarized in Additional file 1. Detailed information on genetic variants for drug targets was shown in Additional files 2, 3.

### Outcome

We focused on the effect of antidiabetic drugs on the risk of stroke, including any stroke (comprising ischemic stroke, intracerebral hemorrhage, and stroke of unknown or undetermined type), any ischemic stroke and intracerebral hemorrhage (ICH).

Genetic association estimates for any stroke, and any ischemic stroke were obtained from the MEGASTROKE GWAS meta-analysis restricted to Europeans (40,585 cases of any stroke, 34,217 cases of ischemic stroke, and 406,111 controls) [13].

Summary statistics for ICH were available from the International Stroke Genetics Consortium (ISGC) GWAS meta-analysis of European descent (1545 cases and 1481 controls) [14]. ICH was defined as a new acute neurological defect. Moreover, the presence of intraparenchymal bleeding was demonstrated using brain imaging (computed tomography or magnetic resonance imaging).

MR study.

Causal effects were estimated with the random-effects inverse variance weighted (IVW) method for mutiple SNPs. For single SNP, Wald Ratio method was used to estimate the causal effects. The random-effects IVW method, the main method of the study, essentially assumed a zero intercept and performed a weighted regression of the SNP-exposure effects with the SNP-outcome effects. For the lowering effects of blood glucose by antidiabetic drugs (sulfonylureas, insulin/insulin analogues, GLP-1 analogues and thiazolidinediones), all estimations were scaled to per 1 mmol/L decrement in blood glucose. For the lowering effects of HbA1c by metformin, the estimations were equivalent to a 6.75 mmol/mol (1.09%) reduction on HbA1c. Multiple tests of three outcomes were adjusted by Bonferroni correction with significance level of P value < 0.017(0.05/3).

Pleiotropy analysis was mainly based on the MR-Egger intercept test and the heterogeneity test using Cochran’s Q statistic [15, 16]. The above analyses showed statistically significant differences when P value < 0.05. Statistical analyses were performed in R version 4.1.2 (TwoSampleMR packages).

### Colocalization analysis

If the targets of antidiabetic drugs were associated with stroke as identified by MR, we further performed the colocalization between blood glucose/HbA1c and stroke within the target gene region.

In colocalization analysis, there are five hypotheses [17]: H0: no association with either trait; H1: association with trait 1, not with trait 2; H2: association with trait 2, not with trait 1: H3: association with trait 1 and trait 2, two independent SNPs; H4: association with trait 1 and trait 2, one shared SNP. Evidence for colocalization was defined as the posterior probability for shared causal variants greater than 0.7 (posterior probability of hypothesis 4 > 0.7). Colocalization analysis was performed using R package Coloc.

### MR validation study using an independent blood glucose GWAS

Through the above analyses, for antidiabetic agents that have effect on the risk of stroke, an independent blood glucose GWAS summary data was selected for further verification.

We chose the summary data of blood glucose GWAS, based on 63,406 individuals of European ancestry from Chen et al. [18]. In the study, individuals were excluded if they had type 1 or type 2 diabetes (defined by physician diagnosis); reported use of diabetes-relevant medications; or had a FG ≥ 7 mmol/L, 2hGlu ≥ 11.1mmol/L, or HbA1c ≥ 6.5%. 2hGlu measures were obtained 120 min after a glucose challenge in an oral glucose tolerance test (OGTT).

We further selected genetic variants as proxies for the blood glucose lowering effects of common antidiabetic drugs. Information on pharmacologically active protein targets and corresponding encoding genes of the antidiabetic agents was obtained from the DrugBank and ChEMBL databases (Additional file 4) [19, 20]. SNPs associated with blood glucose at genome-wide significance level (p < 1 × 10^-5^) and within ± 100 kb windows from the gene region for drug targets of antidiabetic drugs were obtained. For drug targets, SNPs used as proxies were permitted to be in weak linkage disequilibrium (r^2^ < 0.40) with each other to increase the proportion of variance explained by the instrument, maximizing statistical power.

For the antidiabetic drugs, including instruments at moderate to low LD (r^2^ < 0.4), generalized linear regression analyses weighted were applied for the correlation between the instruments, as previously described [21]. Multiple tests of three outcomes were adjusted by Bonferroni correction with significance level of P value < 0.017(0.05/3).

Pleiotropy analysis, heterogeneity test and colocalization analysis were carried out in accordance with the above methods. Statistical analyses were performed in R version 4.1.2 (MendelianRandomization and Coloc packages).

Moreover, to validate our selection of instrumental variables (IVs) from the blood glucose GWAS summary statistics by Chen et al., positive control analyses were performed with type 2 diabetes mellitus, insulin secretion and obesity-related traits as outcomes. Type 2 diabetes mellitus is the original indication of antidiabetic drugs, while TZD and metformin increase insulin sensitivity and sulfonylureas and GLP-1 analogues promote insulin secretion [10]. Obesity is another phenotype that is affected by antidiabetic drugs. A meta-analysis of clinical trials showed that insulin analogues, sulfonylureas, and TZD cause weight gain, and GLP-1 analogues contribute to weight loss [22]. Hence, three obesity related traits, including body mass index (BMI), waist circumference (WC), and hip circumference (HIP), were used as outcomes. Genetically predicted drug effects that showed directional consistency with clinical trial evidence / drug mechanisms were considered to pass the positive control analyses. Information for the GWAS datasets of type 2 diabetes, insulin secretion and obesity related traits used in our study was summarized in Additional file 1 [23–26].

## Results

### Genetic proxies for antidiabetic drugs targets and risk of stroke

#### MR study

**Fig. 2:**
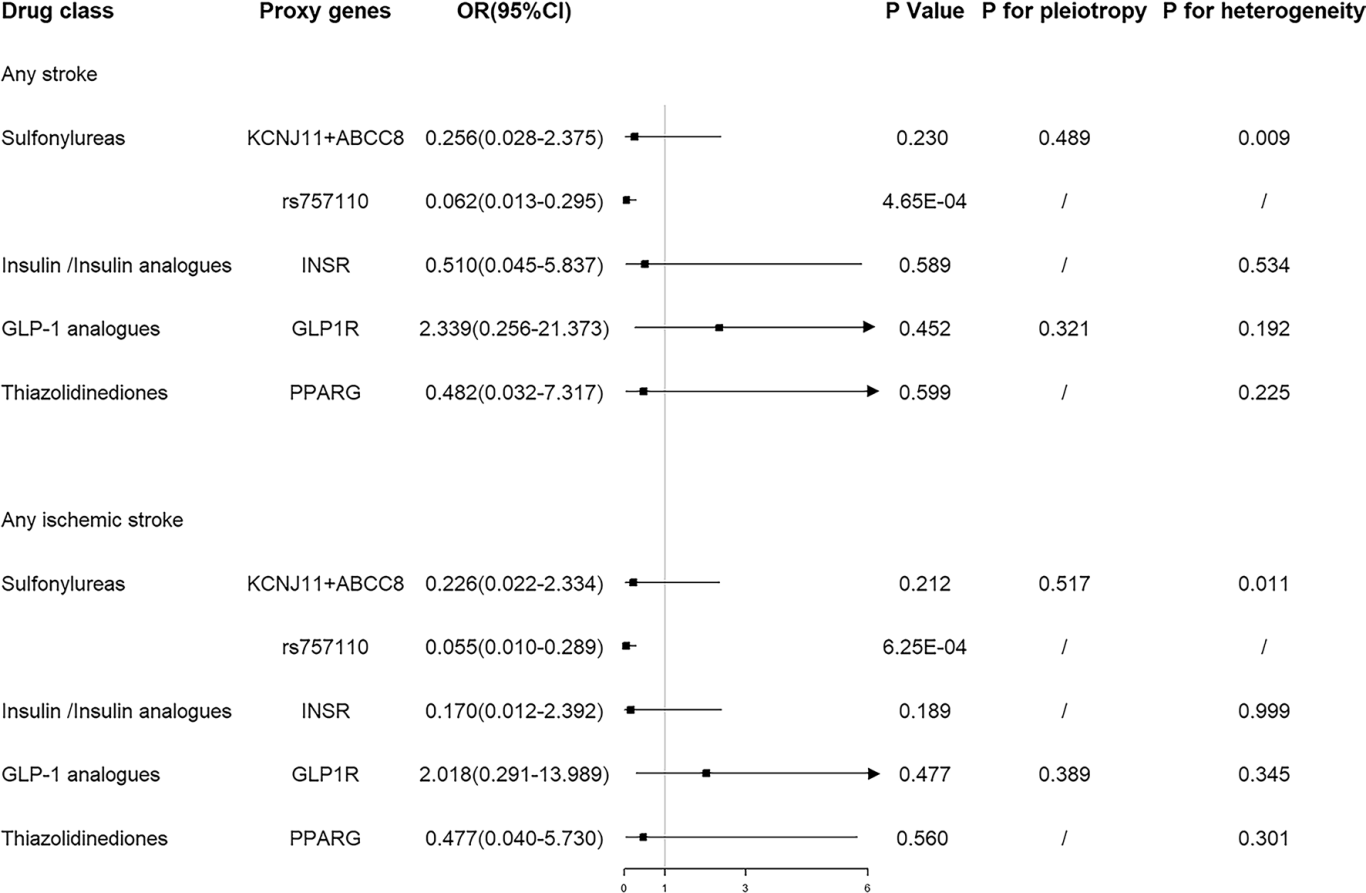
Estimated effects of genetic variants in antidiabetic drugs targets on any stroke and any ischemic stroke

We selected blood glucose-lowering variants in genes encoding drug targets as proxies for the effects of sulfonylureas, insulin/insulin analogues, GLP-1 analogues, and thiazolidinediones. The validated proxy in sulfonylureas targets (rs757110) was associated with reduced risk of any stroke (OR = 0.062, 95% CI 0.013–0.295, P = 4.65 × 10^-4^) and any ischemic stroke (OR = 0.055, 95% CI 0.010–0.289, P = 6.25 × 10^−4^) (Fig. 2). Genetic variants in the targets of sulfonylureas also showed protective effects on any stroke (OR = 0.256, 95% CI 0.028–2.375, P = 0.230) and any ischemic stroke (OR = 0.226, 95% CI 0.022–2.334, P = 0.212), although the effect sizes attenuated toward the null. Genetic variants in the targets of sulfonylureas were not associated with ICH risk (Table 1). Furthermore, genetic variants in the targets of insulin/insulin analogues, GLP-1 analogues, and thiazolidinediones were not associated with the risk of any stroke, any ischemic stroke and ICH (Fig. 2; Table 1).

**Table 1:** Estimated effects of genetic variants in antidiabetic drugs targets on intracerebral hemorrhage

Drug class	Proxy genes	OR	(95% CI)	P value
Sulfonylureas	KCNJ11 + ABCC8	0.781	0.001−434.992	0.939
	rs757110	0.700	2.02 × 10^−4^−2425.925	0.932
Insulin/Insulin analogues	INSR	1,122,841	0.031–4.01 × 10^13^	0.116
GLP-1 analogues	GLP1R	0.029	3.39 × 10^−9^−248751.7	0.664
Thiazolidinediones	PPARG	4.26 × 10^−5^	8.46 × 10^−12^−214.564	0.201

**Fig. 3:**
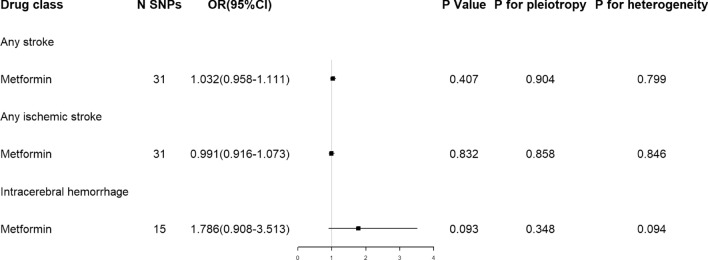
Estimated effects of genetic variants in metformin targets on stroke

For metformin, there was no causal relationship between metformin with the risk of any stroke, any ischemic stroke and ICH (Fig. 3).

There were heterogeneity but no pleiotropy within IVs of sulfonylureas. No heterogeneity and pleiotropy within IVs of other antidiabetic drugs were observed (Figs. 2 and 3).

### Colocalization analysis for sulfonylureas

**Fig. 4:**
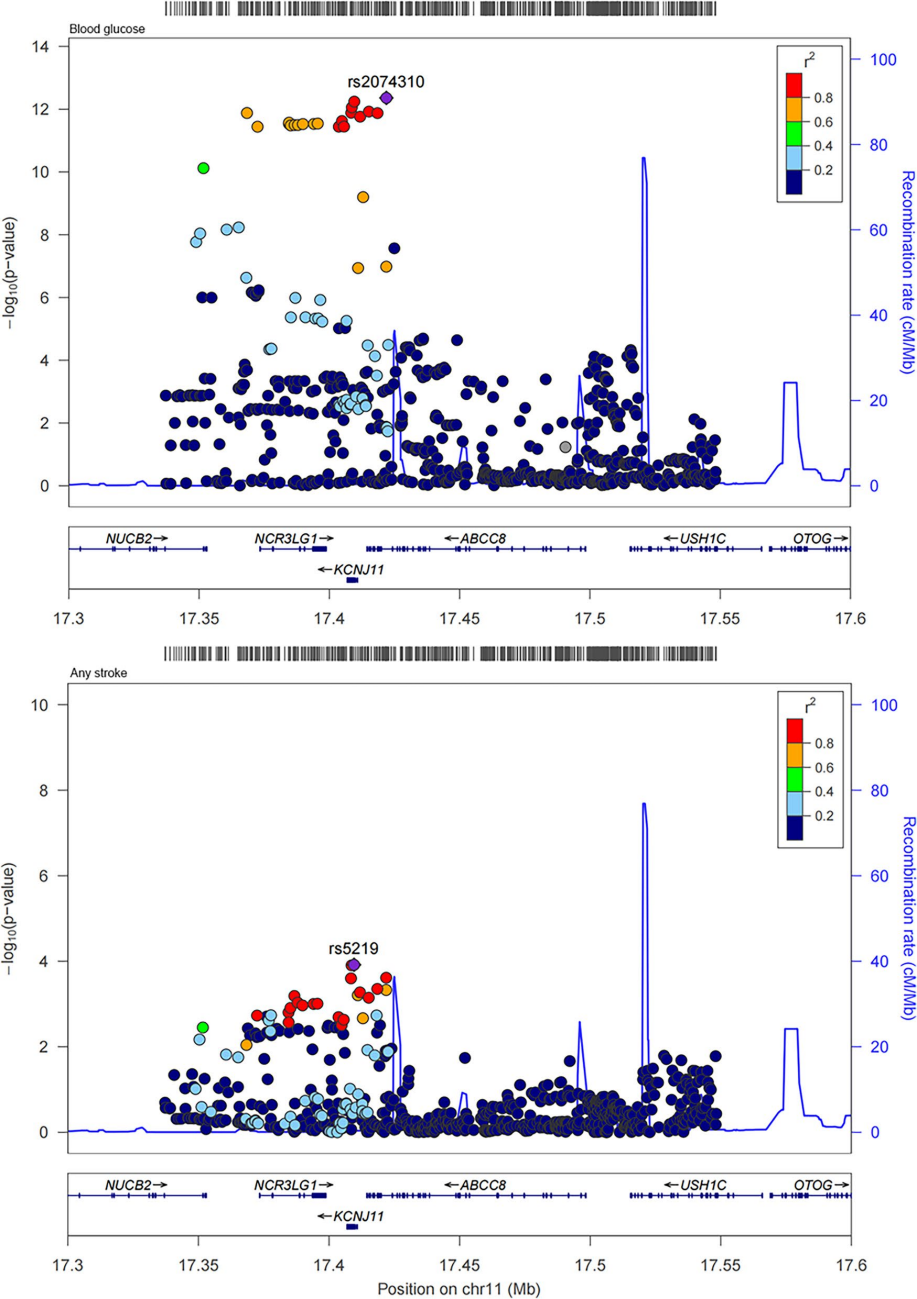
Regional plots for the associations of blood glucose and any stroke within ± 2.5 kb of KCNJ11 and ABCC8

**Fig. 5:**
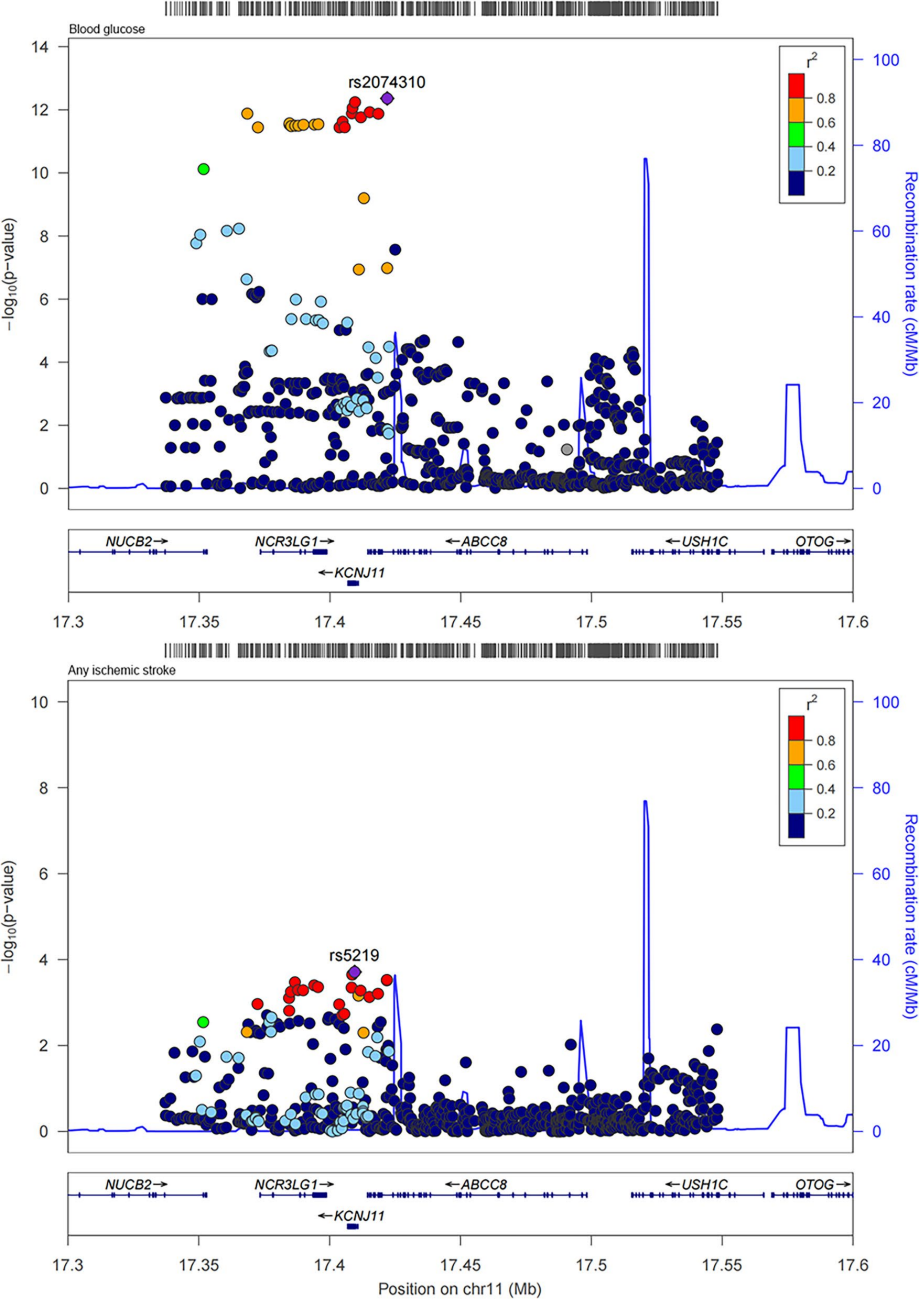
Regional plots for the associations of blood glucose and any ischemic stroke within ± 2.5 kb of KCNJ11 and ABCC8

Through MR analysis, the causal association between sulfonylureas with any stroke, and any ischemic stroke was showed. And then colocalization analysis was performed for sulfonylureas with any stroke, and any ischemic stroke within the drug target encoding genes (± 2500 base pairs of KCNJ11 and ABCC8 ). The results were shown in Table 2. In general, we provided supporting evidence of colocalization between blood glucose with any stroke, and any ischemic stroke within two gene regions of sulfonylureas (posterior probability for a shared causal variant, any stroke: 0.785 in KCNJ11 and 0.739 in ABCC8; any ischemic stroke: 0.750 in KCNJ11 and 0.728 in ABCC8). The regional association plots for the variants within KCNJ11 and ABCC8 of blood glucose with any stroke, and any ischemic stroke were shown in Figs. 4 and 5.

**Table 2:** Colocalization results in the target gene region of sulfonylureas for blood glucose and stroke

Drug class	Stroke	Gene	PP.H0	PP.H1	PP.H2	PP.H3	PP.H4
Sulfonylureas	Any stroke	KCNJ11	1.40e−07	5.19e−09	2.08e−01	6.92e−03	0.785
		ABCC8	3.09e−07	5.93e−09	2.57e−01	4.20e−03	0.739
Sulfonylureas	Any ischemic stroke	KCNJ11	1.62e−07	6.73e−09	2.41e−01	9.25e−03	0.750
		ABCC8	3.22e−07	6.15e−09	2.68e−01	4.40e−03	0.728

The region was defined as ± 2.5 kb of gene region

PP indicates posterior probability

H0: neither trait has a genetic association in the region

H1: only stroke has a genetic association in the region

H2: only blood glucose has a genetic association in the region

H3: both traits are associated, but with different causal variants

H4: both traits are associated and share a single causal variant

### Validation study using an independent blood glucose GWAS

#### MR study

**Fig. 6:**
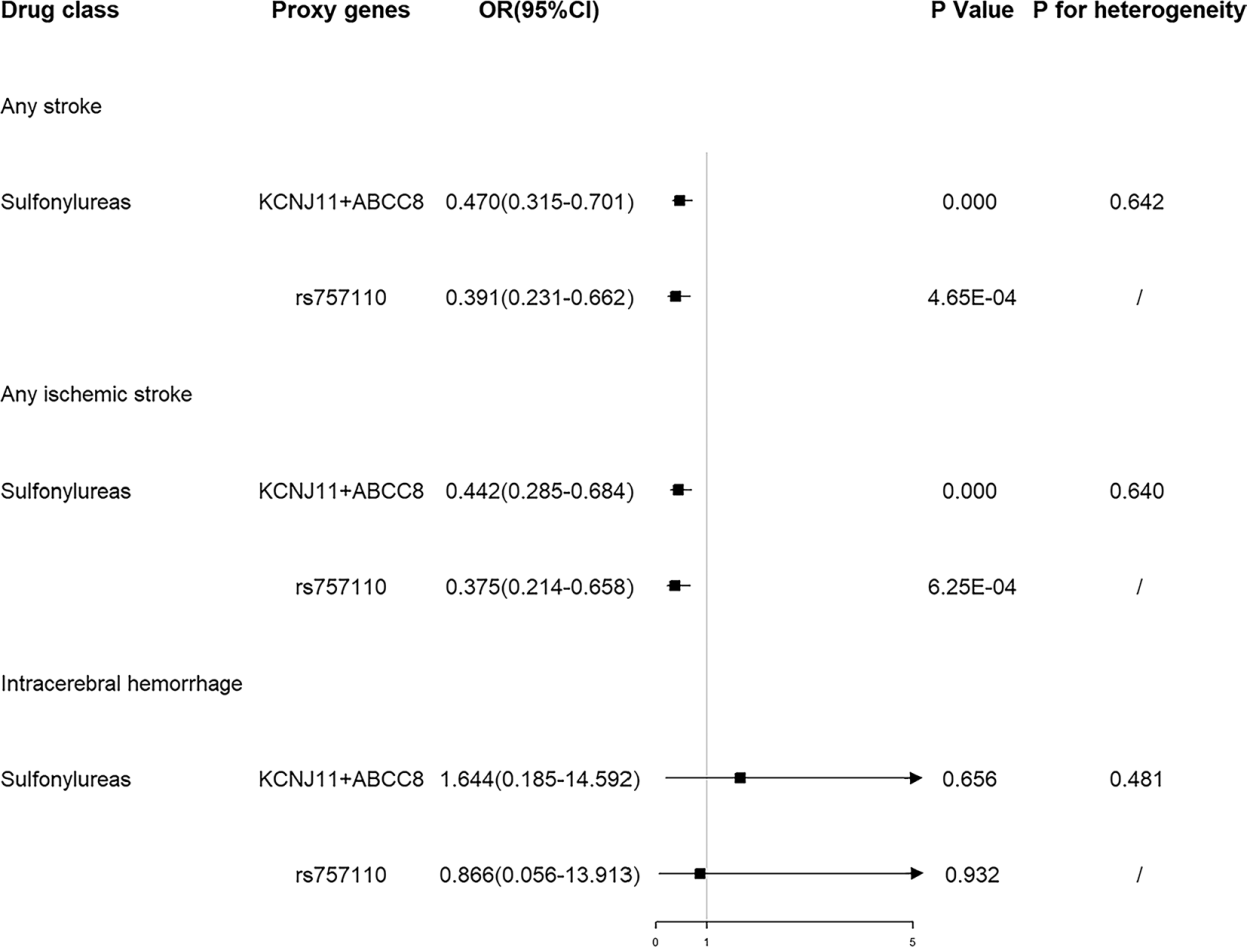
Estimated effects of genetic variants in sulfonylureas targets on stroke using an independent blood glucose GWAS summary data

Through the above analyses, our fingdings suggested that sulfonylureas were associated with stroke risk. Using an independent blood glucose GWAS for validation, 2 variants for sulfonylureas and the validated proxy-rs757110 (Additional file 5) were identified. Genetic variants in sulfonylureas targets had protective effects on risk of any stroke and any ischemic stroke, but not on ICH risk (Fig. 6). The association was consistent in the analysis using the validated proxy, rs757110 (Fig. 6). For all the estimates, no heterogeneity within IVs was detected (Fig. 6).

#### Positive control analyses

**Fig. 7:**
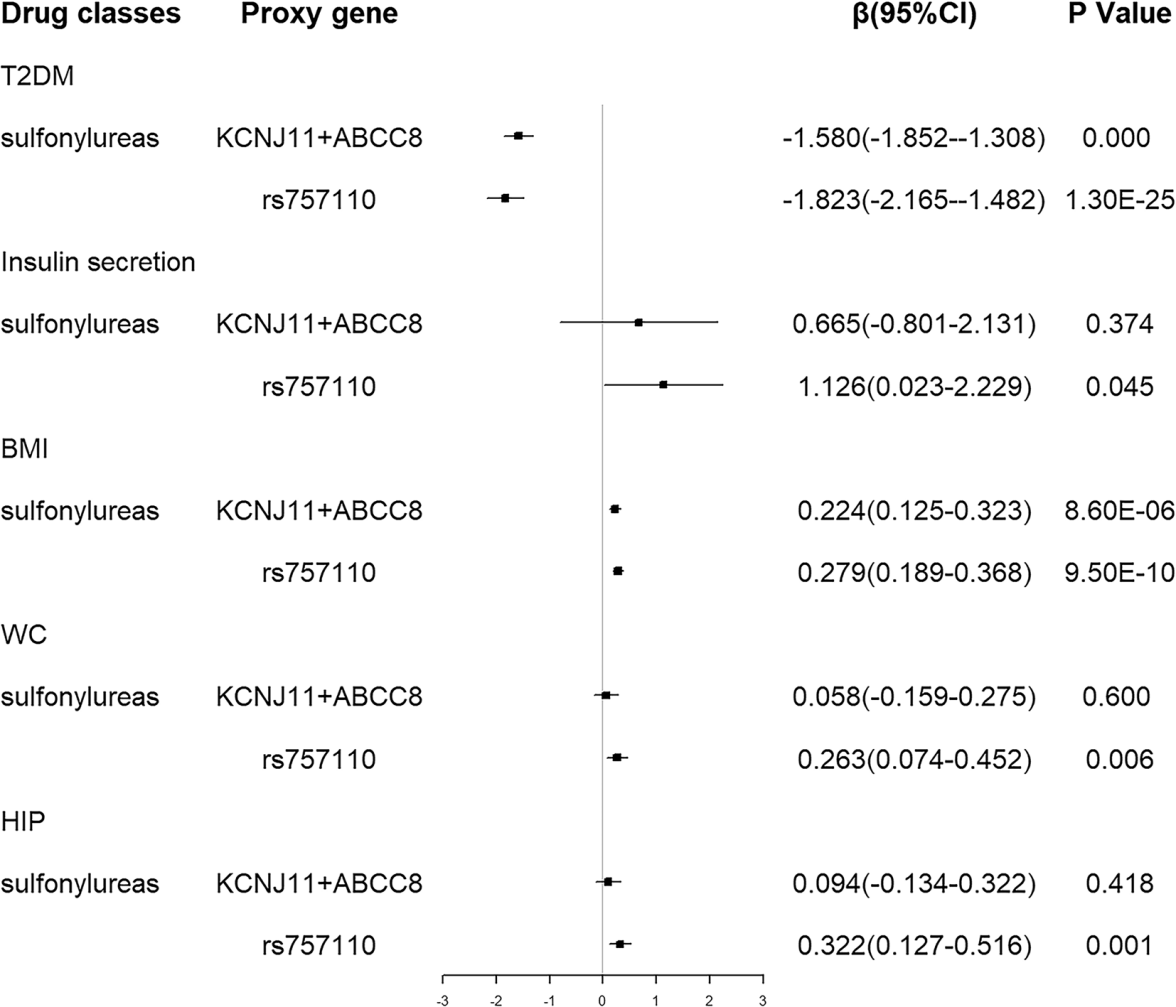
Estimated effects of genetic variants in sulfonylureas targets on glucose metabolism-related traits and obesity-related traits

In order to validate the strength of our IVs, a set of positive control analyses of genetic variants in sulfonylurea targets were conducted. As shown in Fig. 7, genetic variants in the targets of sulfonylureas were associated with decreased risk of type 2 diabetes mellitus and increased BMI. No significant causal relationship between genetic variants in the targets of sulfonylureas and insulin secretion, WC and HIP was found. When looking into the validated proxy, rs757110 was associated with decreased type 2 diabetes mellitus risk and increased insulin secretion, consistent with the drug mechanism of actions. For obesity-related traits, the estimates for rs757110 suggested increment in BMI, WC, and HIP, consistent with evidence from the meta-analysis of clinical trials [22]. Positive control analyses that showed directional consistency with clinical trial evidence / drug mechanisms meant the robustness of our instrumental variables.

### Colocalization analysis for sulfonylureas in validation study

Colocalization analysis for sulfonylureas with any stroke and any ischemic stroke within the drug target encoding genes (± 100 kb of KCNJ11 and ABCC8 ) were performed. The results were shown in Table 3.

**Table 3:** Colocalization results in the target gene region of sulfonylureas for blood glucose and stroke in validation study

Drug class	Stroke	Gene	PP.H0	PP.H1	PP.H2	PP.H3	PP.H4
Sulfonylureas	Any stroke	KCNJ11	4.5e−03	3.0e−04	2.21e−01	1.45e−02	0.760
		ABCC8	4.5e−03	3.0e−04	2.21e−01	1.49e−02	0.759
Sulfonylureas	Any ischemic stroke	KCNJ11	5.0e−03	4.0e−04	2.66e−01	2.0e−02	0.708
		ABCC8	5.0e−03	4.0e−04	2.66e−01	2.1e−02	0.707

The region was defined as ± 100 kb of gene region

PP indicates posterior probability

H0: neither trait has a genetic association in the region

H1: only stroke has a genetic association in the region

H2: only blood glucose has a genetic association in the region

H3: both traits are associated, but with different causal variants

H4: both traits are associated and share a single causal variant

**Fig. 8:**
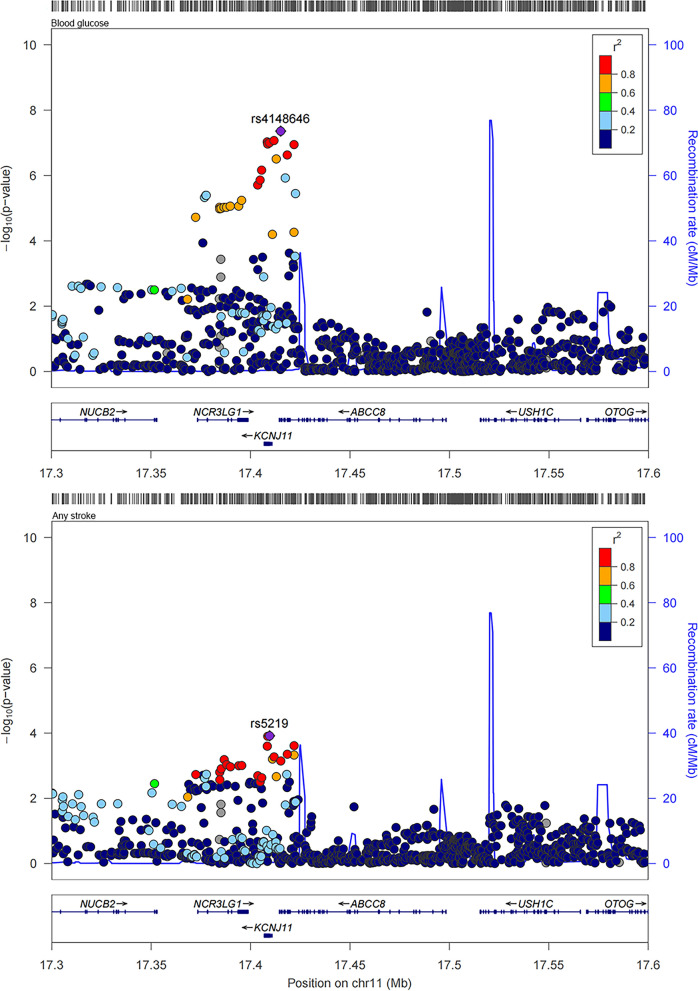
Regional plots for the associations of blood glucose and any stroke within ± 100 kb of KCNJ11 and ABCC8 in validation study

**Fig. 9:**
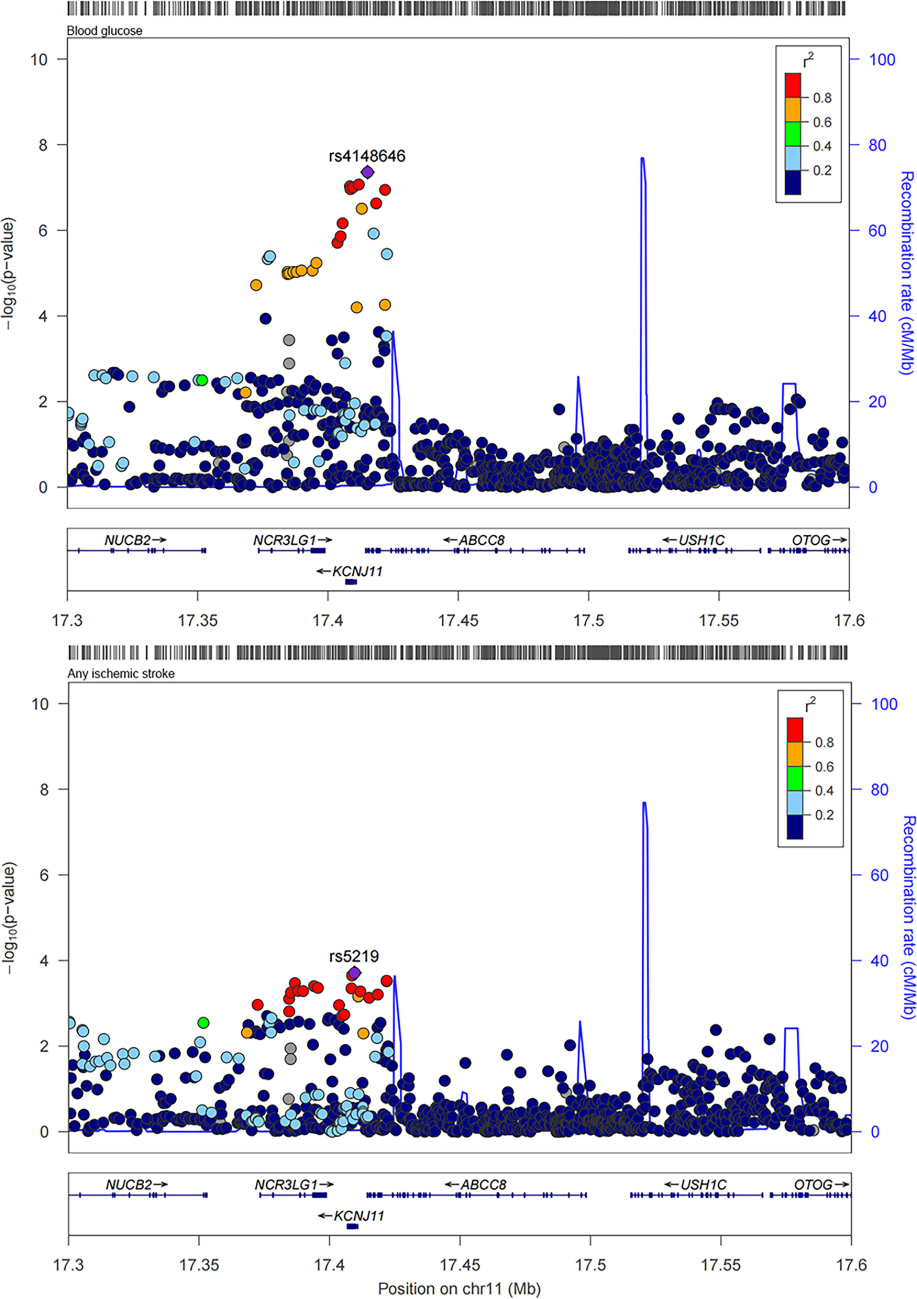
Regional plots for the associations of blood glucose and any ischemic stroke within ± 100 kb of KCNJ11 and ABCC8 in validation study

Similarly, using an independent blood glucose GWAS, we suggested colocalization between blood glucose with any stroke and any ischemic stroke within two gene regions of sulfonylureas (posterior probability for a shared causal variant, any stroke: 0.760 in KCNJ11 and 0.759 in ABCC8; any ischemic stroke: 0.708 in KCNJ11 and 0.707 in ABCC8). The regional association plots for the variants within KCNJ11 and ABCC8 of blood glucose with any stroke, and any ischemic stroke were shown in Figs. 8 and 9.

## Discussion

Using genetic proxies for antidiabetic drugs targets, our results showed sulfonylureas were associated with lower risk of any stroke and any ischemic stroke, but not with ICH. In addition, Bayesian colocalization method was used to eliminate the bias caused by linkage disequilibrium. Using 0.7 as a posterior probability threshold, a high probability of shared casual variants for blood glucose and stroke within the encoding genes for sulfonylureas (KCNJ11 and ABCC8) was suggested. However, there was no causal association between other antidiabetic drugs (including insulin/insulin analogues, GLP-1 analogues, thiazolidinediones and metformin) and stroke risk. It is essential to reduce the burden of stroke by reducing the incidence of stroke in patients with diabetes through the use of antidiabetic drugs. Our study provides supportive suggestions for the priority choice of sulfonylureas as antidiabetic drugs in diabetic patients at high risk of ischemic stroke.

In clinical trials, long-term lowering of blood glucose had modest effect on stroke risk. A meta-analysis summarizing the effects of glucose-lowering drugs on stroke risk showed that glucagon-like peptide-1 receptor agonists and thiazolidinediones reduced stroke risk, while sulfonylureas, dipeptidyl peptidase-4 inhibitors, metformin, sodium-glucose cotransporter 2 inhibitors, α-glucosidase inhibitors, and meglitinides had no significant effect on stroke risk [6].

In our study, using genetic data, among the five antidiabetic drugs, only sulfonylureas reduced the risk of any stroke and any ischemic stroke. Any stroke comprised ischemic stroke, intracerebral hemorrhage, and stroke of unknown or undetermined type. In any stroke GWAS, any stroke patients included nearly 85% ischemic stroke cases, and sulfonylureas had no effect on the risk of hemorrhagic stroke. Therefore, this study suggested that sulfonylureas mainly reduced the risk of ischemic stroke.

Sulfonylureas have been used in the treatment of type 2 diabetes mellitus for more than 60 years due to their good blood glucose-lowering properties and low economic cost. They exhibit principal antidiabetic properties by inhibiting KATP channels and promoting increased insulin secretion from pancreatic beta cells [27, 28]. Previous studies have investigated the effect of sulfonylureas on stroke risk, but the results are rather inconclusive. A recent meta-analysis of 17 trials and 27,705 subjects showed that patients with type 2 diabetes who received sulfonylureas treatment had a higher relative risk of stroke morbidity than those who received comparator drugs [29]. The meta-analysis compared the incidence of stroke in diabetic patients who used sulfonylureas with those who used other drugs, but the results did not prove that sulfonylureas increased the risk of stroke. We suggested that they should compare patients on sulfonylureas treatments with those not on antidiabetic drugs to make the results more convincing.

Using MR, the Copenhagen study showed a risk ratio of 1.48 ( 1.04, 2.11) for ischemic stroke with a glucose increase of 1 mmol/ L. The corresponding risk ratio from the MEGASTROKE study combined with the Copenhagen studies was 1.74 (1.31, 2.18) [6]. They suggested that elevated blood glucose increased the risk of ischemic stroke. Chronic hyperglycemia and advanced glycosylation end-products (AGE) contribute to ‘accelerated atherosclerosis’ through the induction of endothelial damage and cellular dysfunction, leading to cerebral vascular damage [30]. In our study, the reduction in blood glucose through variants in genes encoding targets of sulfonylureas was associated with a significantly lower risk of ischemic troke. Sulfonylureas exhibit blood glucose lowering properties by inhibiting KATP channels and promoting insulin secretion by pancreatic beta cells [27, 28]. The KATP channel in pancreas is a complex composed of four subunits of KCNJ11 gene product Kir6.2 and four subunits of ABCC8 gene product SUR1 [31]. Sulfonylureas bind to SUR1 and block KATP channel. Thus, sulfonylureas might reduce endothelial damage and cellular dysfunction by lowering blood glucose, reducing the risk of ischemic stroke. This study showed that other antidiabetic drugs did not reduce the risk of ischemic stroke by lowering blood glucose, which may be attributed to the different mechanisms of lowering blood glucose. However, the mechanisms underlying the effects of different antidiabetic drugs on stroke risk are worth further investigation.

In addition, the direct effects of sulfonylureas on the brain warrant attention. In the brain, SUR1 serves as a regulatory subunit for both the KATP channel and a nonselective cation channel, NCCa-ATP [32]. Ischemic conditions are reported to trigger opening of both KATP and NCCa-ATP channels. Activation of KATP channels plays a neuroprotective role in ischemia, while sulfonylureas treatment may inhibit the neuroprotective effects of KATP channels and increase the risk of stroke [29]. However, NCCa-ATP channel is crucially involved in development of cerebral edema. The use of sulfonylureas to bind SUR1 and then block NCCa-ATP channel is beneficial for stroke and may provide a new therapeutic approach to stroke [33]. Thus, sulfonylureas might have both beneficial and harmful direct effects on the brain. Given the poor penetration of sulfonylureas across the blood-brain barrier [34], the direct effects of sulfonylureas on the brain in diabetic patients without stroke may be very limited. Whether sulfonylureas therapy reduces or increases the risk of ischemic stroke in these patients by lowering blood glucose becomes even more important.

The present MR study differs from previous observational studies in three key aspects. First, observational studies were done in patients with diabetes, making it impossible to separate the true effects of the drugs from that of diabetes. Meanwhile, the current MR study investigated genetically predicted drug effects in the general population without diabetes. Second, observational studies measured drug use at baseline, but poor monotherapy compliance might lead to contamination. In our MR study, drug compliance was not a major concern because genetic exposure was lifelong. Finally, unmeasured confounding factors may also be an issue in observational studies. Conversely, MR is expected to be less affected by confounders due to random allocation of genetic variants at conception. Thus, our study provided evidence that sulfonylureas reduced the risk of ischemic stroke by lowering blood glucose.

The current MR research has several advantages. First, a set of positive control analyses to verify the strength of our IVs were performed. Second, two sets of validated genetic proxies for sulfonylureas were used, and then a relatively consistent reduced risk of ischemic stroke was observed, further supporting the putative causal relationship between sulfonylureas and ischemic stroke.

The study has several limitations. First, our study could only predict the target effects of antidiabetic drugs. In our MR model, off-target effects that are not exerted through these protein targets cannot be captured. Second, genetically predicted drug effects may differ from therapeutic practice. Exposure to genetic variants begins at birth and continues throughout life. Therefore, our analysis can be interpreted to assess the long-term regulatory role of drug target proteins. In addition, given that the genetic effects are lifelong, our estimates do not reflect the effects of taking antidiabetic drugs during a certain period of life. Third, our results will be difficult to generalize to other ancestral populations because we mainly used the genetic summary data restricted to population of European ancestry. Fourth, since antidiabetic drugs contain few instrumental variables, further studies should be performed in the future if larger blood glucose GWAS data are available. Furthermore, randomized controlled trials of antidiabetic drugs and ischemic stroke risk warrant further implementation.

## Conclusions

In conclusion, our study supported genetic variants of sulfonylureas targets were associated with a lower risk of ischemic stroke. Due to the high incidence of stroke and the widespread use of antidaibetic drugs in diabetic patients, it is important to determine whether the use of antidibetic drugs may reduce the risk of stroke in addition to their glucose-lowering properties. Our results suggested diabetic patients may reduce their risk of ischemic stroke by taking sulfonylureas to lower blood glucose. Further study focused on elucidating the potential mechanisms by which sulfonylureas reduce the risk of ischemic stroke is expected. This study also demonstrates that the MR design may be a promising tool for finding new indications for already approved drugs. This approach can be used as a drug screening tool to provide initial support for drug development.

### Supplementary Information

**Additional file 1:** Descriptive characteristics of the genome-wide association study (GWAS) meta-analyses that were included in this Mendelian randomization study.

**Additional file 2:** Characteristics of instrumental variables for antidiabetic drugs.

**Additional file 3:** Characteristics of instrumental variables for metformin.

**Additional file 4:** The information of antidiabetic drug classes.

**Additional file 5:** Characteristics of instrumental variables for sulfonylureas in validation study.

## Data Availability

This study was based on summary statistics. The GWAS data for 2hGlu, any stroke, ischemic stroke, intracranial hemorrhage, type 2 diabetes, insulin secretion, body mass index, waist circumference, and hip circumference are publicly available. The summary statistics used in this study are outlined in the Additional file 1. Detailed studies of these GWAS can be found in the original research papers.
