# Supplementary material for: Genetically proxied antidiabetic drugs targets and stroke risk
Source: J Transl Med. 2023 Sep 30;21:681. doi: 10.1186/s12967-023-04565-x (PMC10544120; doi:10.1186/s12967-023-04565-x)
Supplement: Supplementary file 1 — Additional file 1: Descriptive characteristics of the genome-wide association study (GWAS) meta-analyses that were included in this Mendelian randomization study. [file 12967_2023_4565_MOESM1_ESM.doc]

Additional file 1. Descriptive characteristics of the genome-wide association study (GWAS) meta-analyses that were included in this Mendelian randomization study

Additional file 2. Characteristics of instrumental variables for antidiabetic drugs

Additional file 3. Characteristics of instrumental variables for metformin

Additional file 4. The information of antidiabetic drug classes

Additional file 5. Characteristics of instrumental variables for sulfonylureas in validation study

Additional file 1 Descriptive characteristics of the genome-wide association study (GWAS) meta-analyses that were included in this Mendelian randomization study

| **Study stage** | **GWAS** | **Phenotype** | **Sample size** | **Ancestry** |
| --- | --- | --- | --- | --- |
| Instrument selection | UK Biobank (Tang et al. analysis)^1^ | Blood glucose | 326,885 individuals | European |
| Instrument selection | UK Biobank (Zheng et al. analysis)^2^ | HbA1c | 415,576 individuals | European |
| Instrument selection for validation | MAGIC^3^ | 2hGlu | 63,406 individuals | European |
| Primary outcome | MEGASTROKE^4^ | Any stroke | 40,585 cases and  406,111 controls | European |
| Primary outcome | MEGASTROKE^4^ | Any ischemic stroke | 34,217 cases and  406,111 controls | European |
| Primary outcome | ISGC^5^ | Intracranial hemorrhage | 1,545 cases and 1,481 controls | European |
| Positive control analyses | Mahajan A et al^6^ | Type 2 diabetes | 74,124 T2D cases and 824,006 controls | European |
| Positive control analyses | Prokopenko I et al^7^ | Insulin secretion | 5,318 non-diabetic participants | European |
| Positive control analyses | GIANT consortium and UK Biobank^8^ | Body mass index | 694,649 individuals | European |
| Positive control analyses | Shungin D et al^9^ | Waist circumference | 224,459 individuals | European |
| Positive control analyses | Shungin D et al^9^ | Hip circumference | 224,459 individuals | European |

HbA1c:glycated hemoglobin; T2D: Type 2 diabetes.

Reference

1. Tang B, Wang Y, Jiang X, et al. Genetic Variation in Targets of Anti-diabetic Drugs and Alzheimer Disease Risk: A Mendelian Randomization Study. Neurology. 2022. doi: 10.1212/wnl.0000000000200771

2. Zheng J, Xu M, Walker V, et al. Evaluating the efficacy and mechanism of metformin targets on reducing Alzheimer's disease risk in the general population: a Mendelian randomisation study. Diabetologia. 2022;65(10):1664-1675. doi: 10.1007/s00125-022-05743-0.

3. Chen J, Spracklen CN, Marenne G, et al. The trans-ancestral genomic architecture of glycemic traits. Nat genet. 2021;53:840-860. doi: 10.1038/s41588-021-00852-9

4. Malik R, Chauhan G, Traylor M, et al. Multiancestry genome-wide association study of 520,000 subjects identifies 32 loci associated with stroke and stroke subtypes. Nat genet. 2018;50:524-537. doi: 10.1038/s41588-018-0058-3

5. Woo D, Falcone GJ, Devan WJ, et al. Meta-analysis of genome-wide association studies identififies 1q22 as a susceptibility locus for intracerebral hemorrhage. Am J Hum Genet. 2014;94:511–521. doi: 10.1016/j.ajhg.2014.02.012.

6. Mahajan A, Taliun D, Thurner M, et al. Fine-mapping type 2 diabetes loci to single-variant resolution using high-density imputation and islet-specific epigenome maps. Nat genet. 2018;50:1505-1513. doi: 10.1038/s41588-018-0241-6

7. Prokopenko I, Poon W, Mägi R, et al. A central role for GRB10 in regulation of islet function in man. PLoS Genet. 2014;10:e1004235. doi: 10.1371/journal.pgen.1004235

8. Pulit SL, Stoneman C, Morris AP, et al. Meta-analysis of genome-wide association studies for body fat distribution in 694 649 individuals of European ancestry. Hum Mol Genet. 2019;28:166-174. doi: 10.1093/hmg/ddy327

9. Shungin D, Winkler TW, Croteau-Chonka DC, et al. New genetic loci link adipose and insulin biology to body fat distribution. Nature*.* 2015;518:187-196. doi: 10.1038/nature14132
