# Supplementary material for: Genetically proxied antidiabetic drugs targets and stroke risk
Source: J Transl Med. 2023 Sep 30;21:681. doi: 10.1186/s12967-023-04565-x (PMC10544120; doi:10.1186/s12967-023-04565-x)
Supplement: Supplementary file 2 — Additional file 2: Characteristics of instrumental variables for antidiabetic drugs. [file 12967_2023_4565_MOESM2_ESM.doc]

**Additional file 2 Characteristics of instrumental variables for antidiabetic drugs**

| **Drug class** | **Proxy gene/variant** | **SNP** | **Effect allele** | **Other**  **allele** | **EAF** | **Beta** | **SE** | **P-value** |
| --- | --- | --- | --- | --- | --- | --- | --- | --- |
| Sulfonylureas | KCNJ11 and ABCC8 | rs117287142 | A | G | 0.987 | 0.0225 | 0.0076 | 0.00299089 |
|  |  | rs2074310 | T | C | 0.356 | 0.0129 | 0.0018 | 4.37E-13 |
|  |  | rs3758953 | A | G | 0.511 | -0.0064 | 0.0017 | 0.000254656 |
|  |  | rs4148630 | G | A | 0.254 | -0.0084 | 0.002 | 2.02E-05 |
|  | rs757110 | rs757110 | C | A | 0.358 | 0.0126 | 0.0018 | 1.33E-12 |
| Insulin analogues | INSR | rs2894553 | C | T | 0.906 | 0.0102 | 0.0029 | 0.000376526 |
|  |  | rs74569625 | A | G | 0.065 | 0.0105 | 0.0036 | 0.00322035 |
| GLP-1 analogues | GLP1R | rs1004280 | A | G | 0.234 | 0.0076 | 0.002 | 0.000150612 |
|  |  | rs10305423 | C | T | 0.971 | 0.0244 | 0.0051 | 1.96E-06 |
|  |  | rs880067 | C | T | 0.737 | -0.0063 | 0.002 | 0.00111116 |
| Thiazolidinediones | PPARG | rs138779828 | G | A | 0.979 | -0.0186 | 0.0059 | 0.00174824 |
|  |  | rs2067819 | G | A | 0.785 | 0.0079 | 0.0021 | 0.000117439 |

SNP: single nucleotide polymorphism; EAF: effect allele frequency; SE: standard error.

Genetic variants for drug targets of sulfonylureas, insulin/insulin analogues, GLP-1 analogues, and thiazolidinediones were selected according to the method reported by Tang et al.^1^
