# Supplementary material for: Genetically proxied antidiabetic drugs targets and stroke risk
Source: J Transl Med. 2023 Sep 30;21:681. doi: 10.1186/s12967-023-04565-x (PMC10544120; doi:10.1186/s12967-023-04565-x)
Supplement: Supplementary file 3 — Additional file 3: Characteristics of instrumental variables for metformin. [file 12967_2023_4565_MOESM3_ESM.doc]

**Additional file 3 Characteristics of instrumental variables for metformin**

| **SNP** | **Effect allele** | **Other**  **allele** | **EAF** | **Beta** | **SE** | **P-value** |
| --- | --- | --- | --- | --- | --- | --- |
| rs10272655 | C | T | 0.793381 | -0.0285906 | 0.0118497 | 1.60E-02 |
| rs1645060 | A | G | 0.0668049 | 0.019421 | 0.0190563 | 3.10E-01 |
| rs3793342 | G | A | 0.855927 | 0.0625106 | 0.0135543 | 4.00E-06 |
| rs72866989 | G | A | 0.989821 | 0.0642601 | 0.0472991 | 1.70E-01 |
| rs1227732 | T | G | 0.192019 | 0.0614894 | 0.0120855 | 3.60E-07 |
| rs1043409 | A | T | 0.94094 | -0.0778956 | 0.0204761 | 1.40E-04 |
| rs117877390 | C | T | 0.973262 | -0.108167 | 0.0305695 | 4.00E-04 |
| rs12969399 | T | G | 0.6415 | -0.0243742 | 0.009942 | 1.40E-02 |
| rs1354034 | T | C | 0.40066 | -0.0614639 | 0.00968259 | 2.20E-10 |
| rs147052086 | G | A | 0.983874 | -0.112877 | 0.0395379 | 4.30E-03 |
| rs150943293 | A | G | 0.981544 | 0.101047 | 0.0354936 | 4.40E-03 |
| rs151128822 | G | A | 0.908493 | 0.0379756 | 0.0164928 | 2.10E-02 |
| rs1532331 | G | T | 0.301764 | -0.0384135 | 0.0104033 | 2.20E-04 |
| rs1809084 | C | T | 0.694106 | -0.0459885 | 0.010309 | 8.20E-06 |
| rs2450122 | T | C | 0.843382 | 0.066012 | 0.0130747 | 4.40E-07 |
| rs2965201 | C | T | 0.168842 | -0.0262833 | 0.0127097 | 3.90E-02 |
| rs3136476 | T | TAGG | 0.842513 | -0.0274894 | 0.0130914 | 3.60E-02 |
| rs4657093 | T | C | 0.869552 | 0.0432839 | 0.0141384 | 2.20E-03 |
| rs4837917 | T | C | 0.7073 | -0.035123 | 0.0104502 | 7.80E-04 |
| rs62180557 | T | C | 0.976835 | 0.0673699 | 0.0316272 | 3.30E-02 |
| rs62372178 | T | C | 0.566611 | 0.0352906 | 0.00963476 | 2.50E-04 |
| rs62383878 | A | C | 0.767891 | 0.055337 | 0.0113057 | 9.90E-07 |
| rs653790 | C | T | 0.755606 | 0.0372501 | 0.0116496 | 1.40E-03 |
| rs6897346 | C | T | 0.802665 | 0.0268523 | 0.0119759 | 2.50E-02 |
| rs73497430 | T | G | 0.753661 | 0.0410355 | 0.0111141 | 2.20E-04 |
| rs77145138 | A | C | 0.989262 | -0.190063 | 0.0472001 | 5.70E-05 |
| rs7788702 | A | G | 0.299063 | 0.0646413 | 0.0103925 | 5.00E-10 |
| rs792699 | C | G | 0.247797 | -0.0194607 | 0.0110919 | 7.90E-02 |
| rs8027626 | T | G | 0.67644 | -0.0376339 | 0.0101741 | 2.20E-04 |
| rs9399137 | T | C | 0.742051 | 0.152049 | 0.0108938 | 2.80E-44 |
| rs9866749 | A | T | 0.294095 | -0.121889 | 0.0106907 | 4.10E-30 |
| rs11889246 | C | A | 0.743242 | 0.0380195 | 0.0108884 | 4.80E-04 |

SNP: single nucleotide polymorphism; EAF: effect allele frequency; SE: standard error.

Genetic variants for drug targets of metformin were selected according to the method reported by Zheng et al.^2^
