# Supplementary material for: Genetically proxied antidiabetic drugs targets and stroke risk
Source: J Transl Med. 2023 Sep 30;21:681. doi: 10.1186/s12967-023-04565-x (PMC10544120; doi:10.1186/s12967-023-04565-x)
Supplement: Supplementary file 4 — Additional file 4: The information of antidiabetic drug classes. [file 12967_2023_4565_MOESM4_ESM.doc]

**Additional file 4 The information of antidiabetic drug classes**

| **Drugs** | **Drug targets** | | **Encoding genes** | | **Gene region**  **(in GRCh37)** |
| --- | --- | --- | --- | --- | --- |
|  | **DrugBank** | **ChEMBL** | **DrugBank** | **ChEMBL** |  |
| Metformin | 5’-AMP-activated  protein kinase subunit  β1 | Mitochondrial complex I (NADH  dehydrogenase) | PRKAB1 | Fifty-eight  encoding  genes | Not included |
|  | Electron transfer  flavoprotein-ubiquinone  oxidoreductase,  mitochondrial | Mitochondrial  glycerol-3-phospate  dehydrogenase | ETFDH | GPD2 |  |
| Insulin/Insulin analogues | Insulin receptor | Insulin receptor | INSR | INSR | Chr19: 7,112,266-7,294,425 |
| Glucagon-like peptide-1  (GLP-1) analogues | Glucagon-like peptide 1 receptor | Glucagon-like peptide 1 receptor | GLP1R | GLP1R | Chr6: 39,016,557-39,059,079 |
| Sulfonylureas (SU) | ATP-sensitive potassium channel | ATP-sensitive potassium channel | KCNJ11  ABCC8 | KCNJ11  ABCC8 | Chr11: 17,386,719-17,410,878  Chr11: 17,414,045-17,498,441 |
| Thiazolidinediones (TZD) | Peroxisome  proliferator-activated  receptor gamma | Peroxisome  proliferator-activated  receptor gamma | PPARG | PPARG | Chr3: 12,328,867-12,475,855 |
