# Supplementary material for: Genetically proxied antidiabetic drugs targets and stroke risk
Source: J Transl Med. 2023 Sep 30;21:681. doi: 10.1186/s12967-023-04565-x (PMC10544120; doi:10.1186/s12967-023-04565-x)
Supplement: Supplementary file 5 — Additional file 5: Characteristics of instrumental variables for sulfonylureas in validation study. [file 12967_2023_4565_MOESM5_ESM.doc]

**Additional file 5 Characteristics of instrumental variables for sulfonylureas in validation study**

| **Drug class** | **Proxy gene/variant** | **SNP** | **Effect allele** | **Other**  **allele** | **EAF** | **Beta** | **SE** | **P-value** |
| --- | --- | --- | --- | --- | --- | --- | --- | --- |
| Sulfonylureas | KCNJ11 and ABCC8 | rs4148646 | C | G | 0.336 | 0.0397 | 0.0078 | 4.39E-08 |
|  |  | rs739689 | A | G | 0.661 | 0.0361 | 0.0084 | 1.19E-06 |
|  | rs757110 | rs757110 | A | C | 0.665 | -0.0373 | 0.0078 | 2.35E-07 |

SNP: single nucleotide polymorphism; EAF: effect allele frequency; SE: standard error.
